# Impact of Lumbar Arthrodesis on Activities of Daily Living in Japanese Patients with Adult Spinal Deformity Using a Novel Questionnaire Focused on Oriental Lifestyle

**DOI:** 10.3390/jcm14155482

**Published:** 2025-08-04

**Authors:** Naobumi Hosogane, Takumi Takeuchi, Kazumasa Konishi, Yosuke Kawano, Masahito Takahashi, Azusa Miyamoto, Atsuko Tachibana, Hitoshi Kono

**Affiliations:** 1 Department of Orthopedic Surgery, Kyorin University, 6-20-2 Shinkawa, Mitaka 181-8611, Tokyo, Japan; kimotako1107@yahoo.co.jp (T.T.); koniayu@hotmail.co.jp (K.K.); kmfcsb23@gmail.com (Y.K.); mtaka@ks.kyorin-u.ac.jp (M.T.); 2 Orthopedic Surgery, Keiyu Orthopedic Hospital, 2267-1 Akouda-cho, Tatebayashi 374-0013, Gunma, Japan; a-miyamoto@ku-kai.or.jp (A.M.); hotchild53@hotmail.co.jp (A.T.); kouno-h@ku-kai.or.jp (H.K.)

**Keywords:** adult spinal deformity, spinal stiffness, Oriental lifestyle

## Abstract

**Background/Objectives:** Correction surgery for adult spinal deformity (ASD) reduces disability but may lead to spinal stiffness. Cultural diversity may also influence how this stiffness affects daily life. We aimed to evaluate the impact of correction surgery on Japanese patients with ASD using a newly developed questionnaire and to clarify how these patients adapt to their living environment postoperatively in response to spinal stiffness. **Methods:** This retrospective study included 74 Japanese patients with operative ASD (mean age: 68.2 ± 7.5 years; fusion involving >5 levels) with a minimum follow-up of 1 year. Difficulties in performing various activities of daily living (ADLs) were assessed using a novel 20-item questionnaire tailored to the Oriental lifestyle. The questionnaire also evaluated lifestyle and environmental changes after surgery. Sagittal and coronal spinal parameters were measured using whole-spine radiographs, and clinical outcomes were assessed using the ODI and SRS-22 scores. **Results:** Coronal and sagittal alignment significantly improved postoperatively. Although the total ADL score remained unchanged, four trunk-bending activities showed significant deterioration. The lower instrumented vertebrae level and pelvic fusion were associated with lower scores in 11 items closely related to trunk bending or the Oriental lifestyle. After surgery, 61% of patients switched from a Japanese-style mattress to a bed, and 72% swapped their low dining table for one with chairs. Both the ODI and SRS-22 scores showed significant postoperative improvements. **Conclusions:** Trunk-bending activities worsened postoperatively in Japanese patients with ASD, especially those who underwent pelvic fusion. Additionally, patients often modified their living environment after surgery to accommodate spinal stiffness.

## 1. Introduction

Patients with adult spinal deformity (ASD) experience a broad spectrum of physical disabilities, including low back pain, trunk imbalance, gait disturbance, and an increased risk of falls, as well as psychological impairment [1,2,3]. Additionally, many studies have reported associations with systemic disorders, such as gastroesophageal reflux disease and pulmonary dysfunction [4,5]. Conservative treatments have limited efficacy in managing these dysfunctions and are unable to correct or prevent the progression of spinal deformity [6]. Corrective surgery has been regarded as a promising intervention for mitigating disability associated with spinal deformities. Correction goals for ASD have been extensively studied, with the most widely recognized target being a pelvic incidence (PI)–lumbar lordosis (LL) mismatch of less than 10° [7]. Moreover, several additional formulas have been proposed in Japan based on population-specific data, thus providing individualized targets for LL correction [8,9,10]. These formulas assist surgeons in preoperative planning by offering patient-specific LL goals to achieve optimal spinal alignment. Therefore, most correction and fusion surgeries for ASD involve the lumbar spine and frequently extend to the ilium to secure a distal foundation, particularly in patients with poor bone quality. Additionally, proximal fixation is often extended to the lower or upper thoracic spine to avoid ending the fusion at the thoracolumbar junction or apex of thoracic kyphosis, which are areas associated with a high risk of junctional complications. Consequently, correction surgery for ASD typically involves an extended range of fusion from the thoracic spine to the pelvis in most patients.

Correction surgery can achieve better coronal and sagittal alignment, which may reduce pain and disability and improve health-related quality of life (HR-QOL) scores, but it may also lead to a loss of spinal mobility. This trade-off is not adequately captured by commonly used HR-QOL measures in ASD evaluation, such as the ODI, SRS-22, or SF-36. To assess limitations in daily activities following lumbar spinal fixation, Hart et al. developed the Lumbar Stiffness Disability Index (LSDI), which is a 10-item patient-reported instrument, and found that LSDI scores showed no significant change 2 years postoperatively, despite significant improvements in other HR-QOL scores [11,12]. Moreover, LSDI scores were not significantly correlated with patient satisfaction, thus suggesting that pan-lumbar arthrodesis may have a limited impact on the activities of daily living (ADLs) in the populations studied.

There is considerable diversity in lifestyles and living environments across ethnicities. In many Asian countries, including Japan, a floor-based lifestyle has traditionally been common and can consist of activities such as having meals while sitting on the floor at a low table, sleeping on floor mattresses, and using squat toilets. Although Westernization has influenced daily habits in these regions, a substantial portion of the population, particularly older adults, continues to maintain the Oriental lifestyle. The impact of lumbar arthrodesis may therefore vary among patients with ASD, depending on their lifestyle. Thus, the LSDI, which was developed in a Western context, may be insufficient for evaluating patients with ASD who follow an Oriental lifestyle. Several studies have used original questionnaires to assess the impact of spinal fusion on ADLs. Togawa et al. reported negative effects on forward-bending activities using a 10-item Disability Score [13]. However, this score includes only seven basic activities and does not reflect those specific to the Oriental lifestyle. Additionally, Ishikawa et al. demonstrated that correction surgery for ASD led to restrictions in ADLs requiring substantial spinal mobility or involving strenuous activity using a 25-item questionnaire that included items related to farm work or activities typical in regions with heavy snowfall [14].

Based on these previous studies, we developed an ADL questionnaire tailored to the Japanese lifestyle, particularly for urban-dwelling patients, including items that assess changes in lifestyle or living environment after surgery. We hypothesized that Japanese patients with ASD experience ADL impairments that are specific to a Japanese lifestyle and are not adequately captured by the LSDI. The aim of this study was to evaluate the impact of correction and fusion surgery on Japanese patients with ASD using this newly developed 20-item questionnaire and to clarify how these patients alter their living environment postoperatively in response to spinal stiffness.

## 2. Materials and Methods

### 2.1. Study Population

This was a retrospective study that included 74 patients with ASD (13 men, 61 women; mean age: 68.2 ± 7.5 years) who underwent spinal correction and fusion surgery with a minimum follow-up of 1 year (mean: 32.1 ± 25.0 months). The inclusion criteria of this study were as follows: age over 40 years, planned correction surgery for ASD involving more than five vertebrae, and a postoperative follow-up period of more than one year. The exclusion criteria were as follows: a history of prior spinal fusion surgery in the thoracic or lumbar spine and having revision surgery to extend the fusion during the follow-up period. The patients were recruited at Keiyu Orthopedic Hospital in Tatebayashi City, located in the Kanto region. All patients were Japanese and had lived in Japan for most of their lives. Based on the SRS-Schwab ASD classification, 45 patients were categorized as type N, 18 as type L, and 11 as type D. None of the patients in this cohort were classified as type T (Table 1). Whole-spine standing coronal and sagittal radiographs were obtained at baseline and at the final follow-up. Spinopelvic parameters were measured, including the lumbar coronal Cobb angle, LL, PI, pelvic tilt, sacral slope, and sagittal vertical axis. To evaluate clinical outcomes, the ODI and SRS-22 scores were collected at baseline and at the final follow-up. The requirement for informed consent was waived because of the retrospective nature of this study and the use of anonymized patient data. This study was conducted in accordance with the Declaration of Helsinki, and the protocol was approved by the Ethics Committee of the institute (Project identification number 3705).

### 2.2. ADL Questionnaire

**Figure 1:**
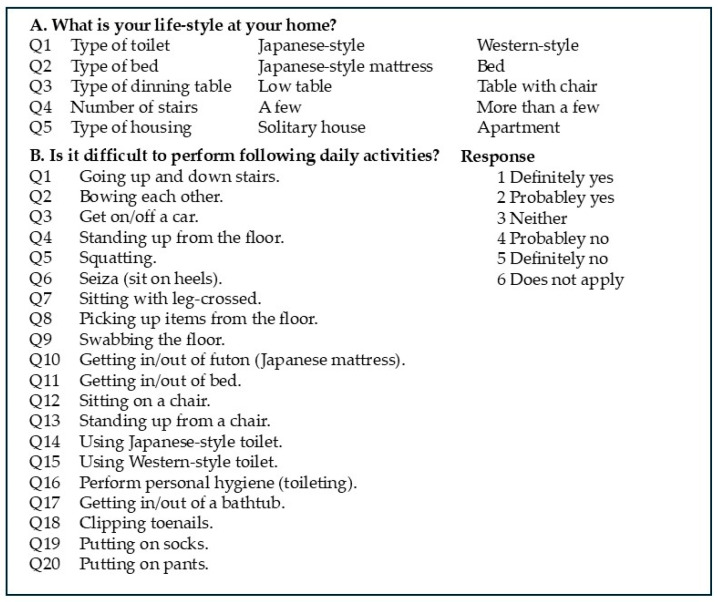
The novel activities of daily living (ADL) questionnaire tailored to an Oriental lifestyle. A: Questions regarding patients’ lifestyle and living environment. B: Questions regarding difficulty in performing 20 ADLs. Patients were asked to choose one of the six responses for each item.

We developed a novel ADL questionnaire to assess the impact of correction surgery on ADLs (Figure 1). The first section of the questionnaire included five items related to patients’ lifestyles and living environments, such as the type of toilet, bed, or dining table used. The second section assessed the difficulty of performing 20 daily activities, including six items modified from the LSDI (Q3, Q8, Q11, Q16, Q17, Q19, and Q20) and six items specifically related to an Oriental lifestyle (Q2, Q4, Q6, Q9, Q10, and Q14). The questionnaire was developed by a multidisciplinary team of spine surgeons and rehabilitation specialists to assess postoperative functional limitations in Japanese patients with ASD. The items were designed to capture both general and culturally specific daily activities, particularly those involving floor-based activities. However, this tool has not yet undergone formal psychometric validation.

Patients responded using a Likert scale by selecting one of the following options for each item: 1. Definitely yes (difficult). 2. Probably yes. 3. Neither. 4. Probably no. 5. Definitely no (not difficult). 6. Does not apply. The total ADL score was calculated by summing the responses, with a maximum possible score of 100. Items for which patients selected “Does not apply” (option 6) were excluded from the analysis, and the total score was calculated as a percentage of the remaining items. We asked patients to complete the ADL questionnaire at their final clinic visit before surgery, together with other HR-QOL assessments such as the ODI and SRS-22. Patients who completed the baseline ADL questionnaire were then asked to complete it again on their follow-up visit.

The total ADL score and individual item scores were compared between baseline and the final follow-up. Additionally, a correlation analysis was performed to examine the relation between each item score and the number of fused segments, as well as the positions of the upper instrumented vertebra (UIV) and lower instrumented vertebra (LIV).

### 2.3. Statistical Analysis

Comparisons of radiographic parameters, HR-QOL scores, total ADL scores, and individual ADL item scores between baseline and the final follow-up were conducted. Prior to the comparison tests, the Shapiro–Wilk test was performed to assess the normality of each variable’s distribution. For variables with a normal distribution, we used parametric tests: paired t-tests for pre- and postoperative comparisons and unpaired t-tests for comparisons between independent groups. For variables that did not follow a normal distribution, we used non-parametric tests: the Wilcoxon signed-rank test for paired data and the Mann–Whitney U test for independent groups. Correlation analyses between each item score and the number of fused segments or the position of the UIV or LIV were performed using Spearman’s rank correlation coefficients. Variables with normal distributions are presented as the mean ± standard deviation (SD), and those without are presented as the median (interquartile range (IQR)). When distributions differed between groups, both were reported as the median (IQR) for consistency with the non-parametric test used. Statistical analyses were performed using SPSS version 24 (IBM Inc. Armonk, NY, USA). For all analyses, a *p*-value < 0.05 was considered statistically significant. In addition to *p*-values, effect sizes were reported to quantify the strength of the associations. Cohen’s d was used for independent and paired t-tests, and effect size r was used for non-parametric comparisons, including the Mann–Whitney U and Wilcoxon signed-rank tests.

## 3. Results

### 3.1. Radiographic Parameters and HR-QOL Scores

The patients had posterior fusion involving a mean of 9.0 ± 2.6 (range: 5–17) vertebrae. The UIV was located in the upper thoracic spine (T2–T5) in 7 patients and at the thoracolumbar junction (T8–L2) in 64 patients. Regarding the LIV, fusion was extended to L2 in 3 patients, L3 in 1, L4 in 2, L5 in 11, S1 in 12, and the ilium in 45. The preoperative LL, PI–LL, and sagittal vertical axis values indicated the presence of a severe sagittal deformity. At the final follow-up, all sagittal and coronal parameters had significantly improved (Table 2). Similarly, the ODI score, all subdomains, and total scores of the SRS-22 showed significant improvements after correction surgery (Table 3).

### 3.2. ADL Score

Although the total ADL score remained unchanged between baseline and the final follow-up, several individual items demonstrated significant changes after spinal fusion. Significant improvements were observed in Q1 (going up and down stairs), Q12 (sitting on a chair), and Q15 (using Western-style toilet). By contrast, the scores significantly worsened for several items, including Q9 (swabbing the floor), Q18 (clipping toenails), Q19 (putting on socks), and Q20 (putting on pants) (Table 4). Correlation analysis was performed between the final follow-up item scores and the number of fused segments, as well as the positions of the UIV and LIV. No significant correlations were found between the item scores and UIV position. However, the number of fused segments had a weak correlation with the score of six items (Q4, Q9, Q10, Q18, Q19, and Q20), and the position of the LIV showed a significant moderate correlation with 11 items (Q4, Q5, Q6, Q7, Q8, Q9, Q10, Q14, Q18, Q19, and Q20) (Table 5).

Subsequently, we evaluated the impact of pelvic fusion on the ADL scores. A total of 45 patients who had fusion to the pelvis (mean age: 69.8 ± 9.9) were compared with the remaining 29 patients (mean age: 65.7 ± 7.7). Although there were no significant differences in any of the 20 ADL items at baseline, patients who underwent pelvic fusion demonstrated significantly lower scores on 11 items at the final follow-up (Q4, Q5, Q6, Q7, Q8, Q9, Q10, Q16, Q18, Q19, and Q20) (Table 6).

### 3.3. Life and Living Environment

Patient lifestyles and living environments were assessed using the first section of the ADL questionnaire. Most patients used Western-style toilets at both baseline and the final follow-up. Among the five patients who initially used a Japanese-style toilet, three switched to a Western-style toilet following surgery. Interestingly, 61% of patients (19 of 31) who had previously used a Japanese-style mattress transitioned to a Western-style bed, and 72% of patients (16 of 22) who had used a Japanese-style low dining table switched to a Western-style dining table with chairs after surgery (Table 7). There were no notable changes in the number of stairs in the patients’ homes or the type of housing after surgery.

## 4. Discussion

In this study, we developed a novel 20-item ADL questionnaire designed to reflect the Japanese lifestyle. This included six items modified from the original LSDI (Questions 3, 8, 11, 16, 17 and 19) and six newly developed items focusing on traditional Japanese activities, such as bowing, standing up from the floor, sitting on one’s heels, swabbing the floor, and using a Japanese-style mattress or toilet (Questions 2, 4, 6, 9, 10 and 14). The aim of this study was to more accurately assess the impact of lumbar stiffness on daily activities pertinent to the Japanese lifestyle.

Our results revealed that the scores of 4 items were significantly worse after correction surgery and that 11 items demonstrated moderate correlations with the position of the LIV, thus suggesting greater disability associated with more distal fixation. In contrast, no significant association was found between ADL scores and the proximal fusion level (UIV). One possible explanation for this is that most patients had fusion ending at the lower thoracic spine, with only a small number having fusion extended to the upper thoracic spine, which may have limited the ability to detect the impact of the UIV position. Another possible explanation is that the caudal extension of fusion may have a greater impact on trunk bending as it restricts movement closer to the pivot point, compared to the fusion extending proximally. The greater impact of fusion to the ilium was supported by our findings, which demonstrated the presence of a significantly higher disability level according to the scores of 11 items for patients with pelvic fixation compared to those without pelvic fixation, despite equivalent baseline scores in all items. As most of the activities of these 11 items involve trunk bending and are closely associated with the Japanese lifestyle, fusion to the pelvis significantly impacts postoperative ADLs in these patients.

Interestingly, our results showed that patients with ASD often modified their living environment after surgery to accommodate spinal stiffness, for example, by switching from a Japanese-style mattress to a Western-style bed or from a low dining table to one with chairs. Such adaptation may explain the maintenance of the postoperative score for items such as Q10 (getting in and out of a Japanese mattress). The lack of change in housing type or the number of stairs in one’s home suggests that the patients found it unnecessary to change their residence postoperatively.

Previous studies from Japan have also reported the impact of spinal fusion on ADLs using original questionnaires. Kimura et al. reported the results of a 21-item questionnaire and demonstrated that patients who underwent three- or four-level fusion experienced more limitations than those with one- or two-level fusion [15]. However, their study population consisted of patients with lumbar degenerative diseases, and the effects of correction surgery for ASD were not specifically assessed. Togawa et al. evaluated the impact of correction surgery on ADLs in patients with ASD, focusing on actions that require bending forward [13]. Their results indicated that patients with ASD experienced persistent postoperative difficulty with forward-bending activities, such as trimming one’s toenails and putting on pants. However, the questionnaire used in this study contained only seven basic activities and did not reflect activities specific to Oriental lifestyles. Another study focusing on ASD patients in non-urban Japan found that correction surgery led to significant restrictions in ADLs associated with an Oriental lifestyle, as well as ADLs involving strenuous field activities, such as farm work or those common in regions with heavy snowfall [14].

Similar studies were conducted in other Asian countries. Choi et al. used a modified LSDI for the Korean lifestyle, which is characterized by floor-based activities and squatting postures [16]. This study found that longer fusion levels and iliac fixation significantly impaired postoperative ADLs, especially in the forward-bending task. A study from China validated the modified Chinese version of the LSDI as a culturally adapted tool for the Chinese lifestyle and demonstrated postoperative deterioration across all items, with several items showing a significant correlation with the number of fused levels [17]. These studies, alongside our own, suggest that spinal fusion has a greater impact on ADLs in Asian populations due to shared floor-based cultural practices. Therefore, it is essential to conduct mutual validation studies of culturally specific ADL questionnaires across Asian countries to allow for a more precise evaluation and comparison of postoperative function in ASD.

By contrast, many studies from North America and Europe using the original LSDI generally concluded that stiffness-related functional impairments were minimal, even in patients with UIV at the upper thoracic spine [12,18]. Daniels et al. also reported no significant difference in postoperative LSDI scores between patients with LIV at L5 and those with LIV at S1 [19]. These discrepancies may arise from the differences in the questionnaires used. The original versions of the LSDI do not include tasks involving floor-based movement, which may result in underestimations of the impact of stiffness in those populations. Moreover, the lower lumbar segments have greater ranges of motion compared to the upper segments, and even the sacroiliac joints possess some degree of mobility. Therefore, fixation extending to more distal levels is likely to result in greater functional limitations. However, in Western lifestyles in which most activities are performed at table height, the clinical impact of such impairments may be limited. In contrast, even modest reductions in mobility can significantly affect daily functioning in floor-based cultures such as Japan’s.

Another potential reason for the cultural differences may be the prevalence of prior spinal fusion. Although our study excluded patients with a history of spinal fusion, a prior multicenter comparison study of ASD patients showed that 22.3% of ASD patients in North America had undergone previous fusion, compared to only 4.2% in Japan [20]. In North American patients, the relatively small changes in ADL scores after corrective surgery may be partly explained by preexisting limitations in mobility due to earlier spinal fusion.

Nevertheless, despite the stiffness-related impairments observed in Asian patients with ASD, disability from lumbar pain or trunk imbalance significantly improved, which was similarly seen in patients with ASD in Western populations, as assessed using the ODI, SRS-22, or SF-36. Although our study demonstrated that fusion to the pelvis had a greater impact on many ADLs, it may benefit patients by providing a more secure distal foundation for achieving better sagittal alignment and is thus indispensable, particularly in older individuals with ASD, severe lumbar degeneration, and flatback deformity. Therefore, obtaining appropriate and specific informed consent, particularly highlighting the possibility of the loss of trunk mobility, is essential before corrective long fusion surgery in patients with ASD with an Oriental lifestyle.

This study had several limitations. First, the sample size was relatively small, and second, due to the retrospective design at a single center, the ADL questionnaire was administered at varying postoperative time points, thus resulting in a wide range of follow-up durations. The potential influence of follow-up duration on ADL scores was not evaluated, and this may have affected the results. Future prospective studies with standardized follow-up protocols are necessary to minimize this bias and better clarify the longitudinal changes in ADLs after corrective surgery for ASD. Third, the impact of the UIV position remains unclear, as most patients had a fusion ending at the lower thoracic spine to the thoracolumbar junction, limiting the variability in UIV levels. Fourth, the ADL questionnaire was administered only to Japanese patients with ASD; its applicability to other ethnic groups, including those in Western countries, remains to be determined. Finally, although the questionnaire was carefully constructed by clinical experts to reflect culturally relevant ADLs, it has not undergone formal validation. Therefore, its psychometric properties, such as reliability and validity, remain to be established. Future studies should address this need.

In conclusion, corrective long spinal fusion surgery significantly impairs specific ADLs in Japanese patients with ASD, particularly those requiring trunk flexion in floor-based lifestyles. Using a culturally adapted 20-item questionnaire, we found that 11 items showed moderate correlation with the LIV, and 11 items showed significant deterioration in patients with pelvic fixation. These findings indicate that distal fusion levels lead to greater postoperative disability in tasks specific to the Oriental lifestyle. Importantly, many patients adjusted their living environments postoperatively to compensate for reduced mobility. These results highlight the need for culturally specific preoperative counseling and the importance of considering lifestyle when planning ASD surgery.

## Figures and Tables

**Table 1:** Patients’ demographics.

Varibles	Value	
Gender (men/women)	13/61	
Mean Age (year)	68.2 ± 7.5	
Follow-Up Period (months)	32.1 (12.0–36.2)	
Number of Fused Segments	9.0 (7.0–10.0)	
SRS-Schwab Curve Type	N	45
	T	0
	L	18
	D	11

Data are expressed as mean ± SD for normally distributed variables and as median (IQR) for non-normally distributed variables.

**Table 2:** Radiographic parameters.

	Baseline	Final Follow-Up
PT (°)	33.7 ± 13.0	29.0 ± 9.7 *
SS (°)	18.0 ± 12.6	24.1 ± 8.6 *
PI (°)	51.1 ± 11.3	53.1 ± 9.2 *
LL (°)	6.0 (−8.0–27.5)	34.6 (26.0–46.3) *
PI-LL (°)	41.3 ± 25.6	16.5 ± 17.6 *
SVA (mm)	103.1 (54.5–151.5)	61.0 (32.8–107.8) *
Lumbar Cobb angle (°)	24.5 (12.4–40.5)	7.5 (2.8–16.1) *

PT: pelvic tilt; SS: sacral slope; PI: pelvic incidence; LL: lumbar lordosis; SVA: sagittal vertical axis. * *p* < 0.05 vs. baseline. Data are expressed as mean ± SD for normally distributed variables and as median (IQR) for non-normally distributed variables. When distributions differed between groups, both were reported as median (IQR).

**Table 3:** Clinical outcomes.

		Baseline	Final Follow-Up
ODI		52.0 ± 17.3	30.1 ± 14.0 *
SRS-22 score	Activity	2.4 (2.0–3.2)	3.4 (2.6–4.0) *
	Pain	3.0 (2.4–3.8)	4.0 (3.2–4.3) *
	Appearance	2.0 ± 0.6	3.4 ± 0.8 *
	Mental health	2.6 (2.0–3.0)	3.2 (3.0–4.0) *
	Satisfaction		3.5 (3.0–4.0)
	Total	2.4 ± 0.6	3.5 ± 0.7 *

* *p* < 0.05 vs. baseline. Data are expressed as mean ± SD for normally distributed variables and as median (IQR) for non-normally distributed variables. When distributions differed between groups, both were reported as median (IQR).

**Table 4:** Change in ADL score after surgery.

	Baseline	Final Follow-Up	Effect Size
Total score	63.0 ± 21.0	60.5 ± 17.0	0.13
Q1	2.0 (2.0–3.0)	3.0 (2.0–4.0) *	0.30
Q2	4.0 (3.0–4.3)	4.0 (3.3–5.0)	0.17
Q3	3.0 (2.0–4.0)	4.0 (2.0–4.0)	0.18
Q4	2.0 (2.0–4.0)	2.0 (2.0–4.0)	0.03
Q5	3.0 (2.0–4.0)	2.0 (1.8–4.0)	0.15
Q6	3.0 (2.0–4.0)	2.0 (1.0–4.0)	0.19
Q7	2.5 (1.0–4.0)	2.0 (1.0–3.5)	0.17
Q8	3.0 (2.0–4.0)	2.0 (2.0–4.0)	0.12
Q9	2.0 (2.0–4.0)	2.0 (1.0–3.0) *	0.36
Q10	3.0 (2.0–4.0)	3.0 (2.0–4.0)	0.03
Q11	3.0 (2.0–4.0)	4.0 (2.0–4.0)	0.19
Q12	4.0 (3.0–4.0)	4.0 (4.0–5.0) *	0.32
Q13	4.0 (3.0–4.0)	4.0 (3.0–5.0)	0.21
Q14	2.0 (1.0–4.0)	2.0 (1.0–3.0)	0.17
Q15	4.0 (3.0–5.0)	4.0 (4.0–5.0) *	0.28
Q16	4.0 (3.0–5.0)	3.0 (3.0–4.8)	0.23
Q17	4.0 (3.0–4.0)	3.0 (2.0–4.0)	0.16
Q18	3.0 (2.0–4.0)	2.0 (1.0–3.0) *	0.46
Q19	3.5 (2.0–4.0)	2.0 (2.0–4.0) *	0.40
Q20	4.0 (2.0–4.0)	3.0 (2.0–4.0) *	0.26

* *p* < 0.05 vs. baseline. Data are expressed as mean ± SD for normally distributed variables and as median (IQR) for non-normally distributed variables. Effect sizes are reported as Cohen’s d for t-tests and effect size r for non-parametric tests.

**Table 5:** The correlation between item scores at the final follow-up and the surgical variables.

**Question**	**Fusion segs**	**UIV**	**LIV**
1 Going up/down stairs	0.06	−0.07	−0.05
2 Bowing	−0.11	0.07	−0.16
3 Get on/off a car	−0.19	0.10	−0.21
4 Standing up from the floor	−0.24 *	0.07	−0.59 *
5 Squatting	−0.22	0.03	−0.57 *
6 Seiza	−0.13	0.07	−0.39 *
7 Sitting leg-crossed	−0.18	0.03	−0.34 *
8 Picking up items from the floor	−0.12	−0.04	−0.33 *
9 Swabbing the floor	−0.25 *	0.08	−0.54 *
10 Getting in/out of futon	−031 *	0.14	−0.40 *
11 Getting in/out of bed	0.02	−0.03	−0.03
12 Sitting on a chair	0.05	0.02	−0.03
13 Standing up from a chair	0.13	−0.127	−0.09
14 Using a Japanese-style toilet	−0.23	0.16	−0.27 *
15 Using a Western-style toilet	0.10	−0.07	0.01
16 Personal hygiene (toilet)	0.03	−0.13	−0.21
17 Getting in/out of a bathtub	−0.054	0.04	−0.19
18 Clipping toenails	−0.29 *	0.16	−0.50 *
19 Putting on socks	−0.34 *	0.12	−0.48 *
20 Putting on pants	−0.24 *	0.05	−0.44 *

UIV: upper instrumented vertebra. LIV: lower instrumented vertebra. * *p* < 0.05 with Spearman’s rank correlation analysis.

**Table 6:** Pelvic fixation and ADL score for each item.

	Baseline		Final Follow-Up		Effect Size
	Pelvic Fixation (+)	Pelvic Fixation (−)	Pelvic Fixation (+)	Pelvic Fixation (−)	
Q1	1.5 (1.0–3.0)	2.0 (2.0–3.0)	3.0 (2.0–3.8)	3.0 (2.0–4.0)	0.06
Q2	2.5 (2.0–4.3)	4.0 (2.8–4.3)	4.0 (3.3–4.8)	4.0 (3.0–4.0)	0.22
Q3	2.0 (1.0–4.0)	3.0 (2.0–4.0)	4.0 (3.1–4.8)	4.0 (2.8–4.0)	0.23
Q4	2.0 (1.0–3.3)	2.0 (1.0–3.0)	3.0 (3.0–4.0)	2.0 (1.8–2.3) *	0.59
Q5	2.0 (1.0–3.3)	2.0 (1.8–4.0)	3.0 (2.0–4.0)	2.0 (1.0–2.0) *	0.57
Q6	2.0 (1.0–3.3)	2.0 (1.0–3.0)	2.0 (1.3–4.0)	2.0 (1.0–3.0) *	0.42
Q7	2.0 (1.0–4.0)	2.0 (1.0–4.0)	2.5 (1.3–4.0)	2.0 (1.0–2.5) *	0.32
Q8	2.0 (1.0–4.0)	2.0 (1.8–4.0)	3.0 (2.0–4.0)	2.0 (1.8–4.0) *	0.35
Q9	2.0 (1.0–4.0)	3.0 (1.8–4.0)	3.0 (2.0–3.0)	2.0 (1.0–2.0) *	0.53
Q10	1.5 (1.0–3.0)	3.0 (2.0–4.0)	3.0 (3.0–4.0)	2.0 (1.8–4.0) *	0.34
Q11	2.0 (1.0–3.0)	3.5 (2.0–4.0)	4.0 (3.0–4.0)	4.0 (2.0–4.0)	0.03
Q12	2.0 (1.8–4.0)	4.0 (3.0–4.0)	4.0 (3.3–5.0)	4.0 (3.8–5.0)	0.08
Q13	2.5 (1.0–4.0)	4.0 (2.8–4.0)	4.0 (3.0–4.0)	4.0 (2.8–4.3)	0.11
Q14	1.5 (1.0–2.0)	2.5 (1.0–3.3)	2.0 (1.0–2.8)	2.0 (1.0–2.0)	0.19
Q15	3.0 (2.0–4.3)	4.0 (4.0–5.0)	4.0 (4.0–5.0)	4.0 (4.0–5.0)	0.04
Q16	3.5 (2.0–5.0)	4.0 (2.8–5.0)	4.0 (3.3–4.8)	3.0 (2.0–4.3) *	0.27
Q17	3.0 (1.8–4.3)	4.0 (2.0–5.0)	3.0 (3.0–4.0)	3.0 (2.0–4.0)	0.23
Q18	2.5 (1.0–4.0)	4.0 (2.0–4.3)	4.0 (2.0–4.0)	2.0 (1.0–2.0) *	0.56
Q19	2.0 (1.0–3.3)	3.5 (2.0–4.3)	3.5 (2.3–4.0)	2.0 (1.0–3.0) *	0.52
Q20	2.5 (1.0–3.3)	4.0 (2.0–4.0)	4.0 (3.0–4.0)	3.0 (1.0–4.0) *	0.47

* *p* < 0.05 vs. pelvic fixation (+). Data are expressed as median (IQR). Effect sizes are reported as effect size *r* for non-parametric tests.

**Table 7:** A comparison of lifestyles and living environments at the baseline and the final follow-up.

		Baseline (%)	Final Follow-Up (%)
Type of toilet	Western-style toilet	93.2	97.3
	Japanese-style toilet	6.8	2.7
Type of bed	Western-style bed	58.1	83.6
	Japanese-style mattress	41.9	16.4
Type of table	Dining table with chairs	70.3	91.8
	Japanese-style low dining table	29.7	8.2
Stairs in the patient’s home	A few steps	61.1	63.4
	More than a few steps	38.9	36.6
Type of housing	Detached house	86.3	87.5
	Apartment	13.7	12.5

## Data Availability

The data are available upon reasonable request.

## Abbreviations

The following abbreviations are used in this manuscript:

ASD	Adult spinal deformity
PI	Pelvic incidence
LL	Lumbar lordosis
HR-QOL	Health-related quality of life
LSDI	Lumbar Stiffness Disability Index
ADLs	Activities of daily living
UIV	Upper instrumented vertebra
LIV	Lower instrumented vertebra
